# Unveiling transcription factor regulation and differential co-expression genes in Duchenne muscular dystrophy

**DOI:** 10.1186/s13000-014-0210-z

**Published:** 2014-10-23

**Authors:** Lijun Tian, Junhua Cao, Xingqiang Deng, Chuanling Zhang, Tong Qian, Xianxiang Song, Baoshan Huang

**Affiliations:** Clinical laboratory, Xuzhou Children’s Hospital, No. 18 Sudibei Road, Xuzhou, Jiangsu Province China

**Keywords:** Duchenne muscular dystrophy, Differential co-expression analysis, Transcription factor

## Abstract

**Background:**

Gene expression analysis is powerful for investigating the underlying mechanisms of Duchenne muscular dystrophy (DMD). Previous studies mainly neglected co-expression or transcription factor (TF) information. Here we integrated TF information into differential co-expression analysis (DCEA) to explore new understandings of DMD pathogenesis.

**Methods:**

Using two microarray datasets from Gene Expression Omnibus (GEO) database, we firstly detected differentially expressed genes (DEGs) and pathways enriched with DEGs. Secondly, we constructed differentially regulated networks to integrate the TF-to-target information and the differential co-expression genes.

**Results:**

A total of 454 DEGs were detected and both KEGG pathway and ingenuity pathway analysis revealed that pathways enriched with aberrantly regulated genes are mostly involved in the immune response processes. DCEA results generated 610 pairs of DEGs regulated by at least one common TF, including 78 pairs of co-expressed DEGs. A network was constructed to illustrate their relationships and a subnetwork for DMD related molecules was constructed to show genes and TFs that may play important roles in the secondary changes of DMD. Among the DEGs which shared TFs with *DMD*, six genes were co-expressed with *DMD*, including *ATP1A2*, *C1QB*, *MYOF*, *SAT1*, *TRIP10*, and *IFI6*.

**Conclusion:**

Our results may provide a new understanding of DMD and contribute potential targets for future therapeutic tests.

**Virtual slides:**

The virtual slide(s) for this article can be found here: http://www.diagnosticpathology.diagnomx.eu/vs/13000_2014_210

## Background

Duchenne muscular dystrophy (DMD) is a severe and progressive inherited neuromuscular disease characterized by muscle fiber degeneration and central nervous system disorders that affects 1 in 3600–6000 live male births [1]. This disease is caused by mutations or dysregulation of the X-linked dystrophin gene. Absence of or defects of dystrophin protein result in disruption of the dystrophin-associated protein complex (DAPC), resulting in chronic inflammation and progressive muscle degeneration [2].

Although mutations of the DMD protein have been identified to be primarily responsible for the pathology, comprehensive understanding of the downstream mechanisms due to dystrophin absence is still lacking. Secondary changes in DMD involve calcium homeostasis [3], nitric oxide synthase [4], inflammation [5] and mast cell degranulation [6], suggesting that the pathological process of DMD is highly complicated. Gene expression profile analysis is a powerful strategy to investigate the pathophysiological mechanisms of DMD. Several gene expression profiling studies [7-11] have been performed earlier, providing insights into the pathology of DMD. However, most of them focused on expression level changes of individual genes, without considering the differences in gene interconnections. Since the majority of proteins function with other proteins, differential co-expression analysis (DCEA) would provide a better understanding of the mechanism underlying DMD progression. The rationale of DCEA is that changes in gene co-expression patterns between two disease statuses (case and control) provide hints regarding the disrupted regulatory relationships in patients. Therefore, integrate the information of Transcription factors (TFs) into DCEA may detect the regulatory causes of observed co-expression changes since TFs can regulate gene expression through binding the cis-elements in the target genes’ promoter regions. TFs are reported to be important in both normal and disease states [12]. Investigation of TF activities in mdx mice has proposed several TFs involved in DMD pathogenesis [13]. Thus, integrative analysis of TFs and target differential co-expression genes may provide better understanding on molecular mechanism of the secondary changes of DMD.

In this study, using microarray gene expression data collected from the Gene Expression Omnibus (GEO) database, we carried out an integrative bioinformatics analysis. Firstly, differentially expressed genes (DEGs) were detected and pathways enriched with DEGs were acquired. Secondly, differentially regulated link networks were constructed to integrate the TF-to-target information and the differential co-expression genes. Our results may provide new understanding of DMD and new targets for future therapeutic tests.

## Methods

### Microarray data

We used two datasets (GSE6011 and GSE3307) from the GEO (http://www.ncbi.nlm.nih.gov/geo/) database. For further combined analysis, we only selected samples generated with the platform GPL96: [HG-U133A] Affymetrix Human Genome U133A Array.

### Detection of differentially expressed genes (DEGs)

Raw data including simple omnibus format in text (SOFT) family files and CEL files of all samples were downloaded. The CEL files contained the expression information for each probe. Robust Multiarray Analysis (RMA) [14] was used for raw intensity values normalization: firstly, background noise and processing artifacts were neutralized by model-based background correction; secondly, expression values were set to a common scale by quantile normalization; thirdly, expression value of each probe was generated by an iterative median polishing procedure. The resulting log2-transformed RMA expression value was then used for detecting DEGs. Statistical *t* test was used to identify DEGs and the Benjamini and Hochberg procedure [15] were carried out to correct the multiple testing problems. The threshold of significant DEGs was set as *P* <0.01. Up- or down-regulation of the DEGs were determined according to the fold-change. All of the above procedures were performed using the R software (v3.0.3) with BioConductor and limma packages (3.12.1) and libraries [16].

### Functional analysis

Differentially expressed probes were annotated based on the SOFT files. All genes were then mapped to the Kyoto En-cyclopedia of Genes and Genomes (KEGG) pathways (http://www.genome.jp/kegg/) database. The hyper geometric distribution test was used to identify pathways significantly enriched with DEGs. In addition to KEGG pathway analysis, DEGs were also uploaded into the Ingenuity Pathway Analysis (IPA) software (Ingenuity® Systems, http://www.ingenuity.com) and was associated with the canonical pathways, molecular and cellular functions, diseases and disorders in the Ingenuity Knowledge Base. Fisher’s exact test was implemented to assess the significance of the associations between DEGs and canonical pathways, functions, or diseases. For canonical pathways, a ratio was also computed between the number of DEGs and the total number of molecules in the pathway.

### DCEA and TF-to-target analysis

The DEGs were subjected to DCEA and TF-to-target analysis using the DCGL package (v2.0) [17] in the R software. Human TF-to-target library was incorporated into the package to identify differentially regulated genes and links. A total of 215 human TFs and 16,863 targets were included in the package. Differentially co-expressed genes (DCGs) were firstly identified and a network was constructed with DCGs and their shared TFs. Since dysregulation of *DMD* gene is the primary cause of the disease, we also constructed a subnetwork surrounding the predefined gene *DMD*.

## Results

With the two downloaded datasets (GSE6011 and GSE3307), expression profile of 27 DMD patients (22 from GSE6011 and 5 from GSE3307) and 14 healthy controls were included in this study. Dystrophin protein was absent in all patients [9,10]. A total of 454 genes were detected to be DEGs, including 284 up regulated genes and 170 down regulated ones. KEGG pathway analysis revealed that aberrantly regulated genes were enriched in 10 pathways (Table 1). Most of these pathways (7/10) are involved in the inflammatory/immune response process, such as the immune system, immune disease and infectious disease. In addition, a DMD related pathway, the viral myocarditis pathway, was included. The other two pathways are Cell adhesion molecules (CAMs) and Focal adhesion.

**Table 1:** **KEGG pathways enriched with differentially expressed genes**

**ID**	**Pathway description**	**Pathway class**	***P*** **-value**
hsa05322	Systemic lupus erythematosus	Immune diseases	7.40E-05
hsa05416	Viral myocarditis	Cardiovascular Diseases	1.60E-04
hsa04514	Cell adhesion molecules (CAMs)	Signaling Molecules and Interaction	4.00E-03
hsa05310	Asthma	Immune Diseases	1.00E-02
hsa04510	Focal adhesion	Cell Communication	1.00E-02
hsa05130	Pathogenic Escherichia coli infection	Infectious Diseases	1.10E-02
hsa04672	Intestinal immune network for IgA production	Immune system	1.90E-02
hsa05330	Allograft rejection	Immune Diseases	1.70E-02
hsa04612	Antigen processing and presentation	Immune System	2.20E-02
hsa05332	Graft-versus-host disease	Immune Diseases	2.10E-02

**Figure 1:**
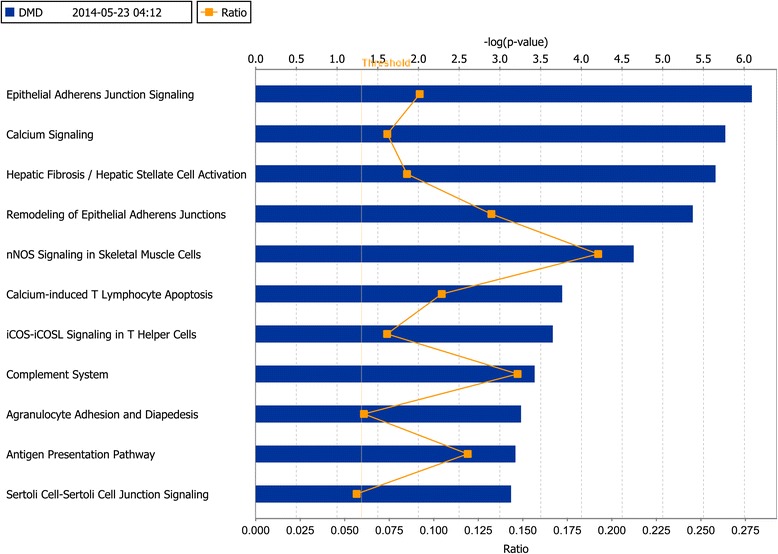
**Ingenuity pathway analysis: the most significant canonical pathways in which differently expressed genes (DEGs) were enriched.**

The IPA results of canonical pathways showed that the DEGs were enriched for epithelial adherens junction signaling, calcium signaling, nNOS signaling in skeletal muscle cells and some additional pathways (Figure 1). Consistent with the KEGG pathway analysis, aberrantly regulated genes were enriched in immune system pathways, such as calcium-induced T lymphocyte apoptosis, iCOS-iCOSL signaling in T helper cells, complement system, and antigen presentation pathway (Figure 1). Diseases and Disorders analysis (Table 2) revealed that “Neurological Disease” (*P* = 3.98E-15–1.20E-02) was the top disease affected by these DEGs followed by “Skeletal and Muscular Disorders” (*P* = 2.66E-13–1.07E-02). “Cellular Growth and Proliferation” (*P* = 1.38E-14–1.12E-02) was the top biological function mediated by these DEGs (Table 2).

**Table 2:** **Ingenuity pathway analysis: diseases and functions related to differentially expressed genes**

**Name**	***P*** **-value**	**# Molecules**
**Diseases and Disorders**		
Neurological disease	3.98E-15–1.20E-02	133
Skeletal and muscular disorders	2.66E-13–1.07E-02	118
Cardiovascular disease	5.89E-13–9.41E-03	75
Cancer	9.06E-11–1.14E-02	293
Psychological disorders	3.24E-10–1.13E-02	92
**Molecular and Cellular functions**		
Cellular growth and proliferation	1.38E-14–1.12E-02	136
Cell death and survival	1.24E-13–1.20E-02	128
Cellular development	8.68E-08–1.12E-02	106
Cellular movement	1.15E-07–1.20E-02	70
Cellular assembly and organization	6.30E-06–9.41E-03	73

**Figure 2:**
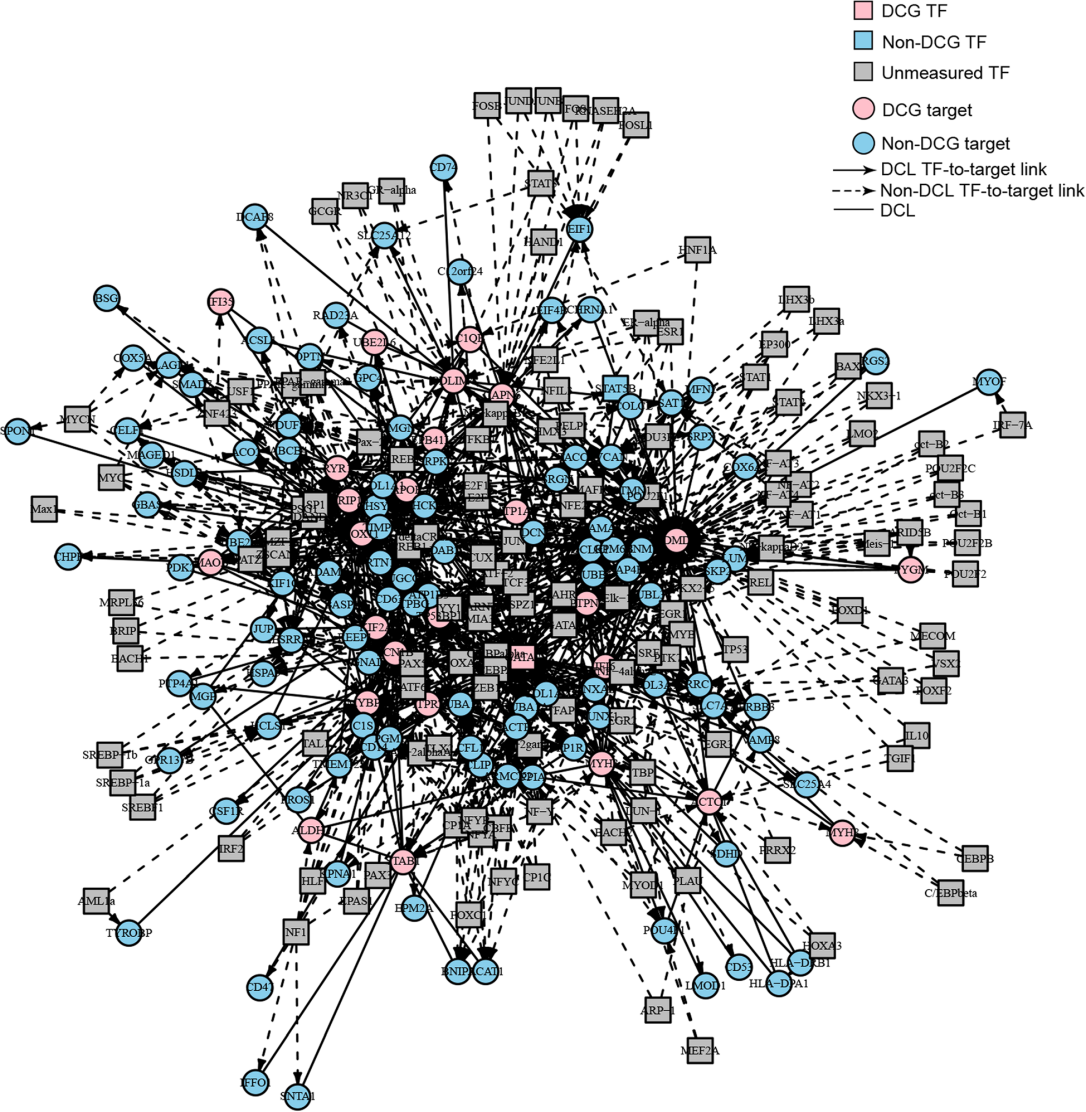
**Transcription factor (TF) regulation network for the 78 pairs of co-expressed differently expressed genes (DEGs).** DCG: differential co-expression genes; DCL: links between DCGs.

**Figure 3:**
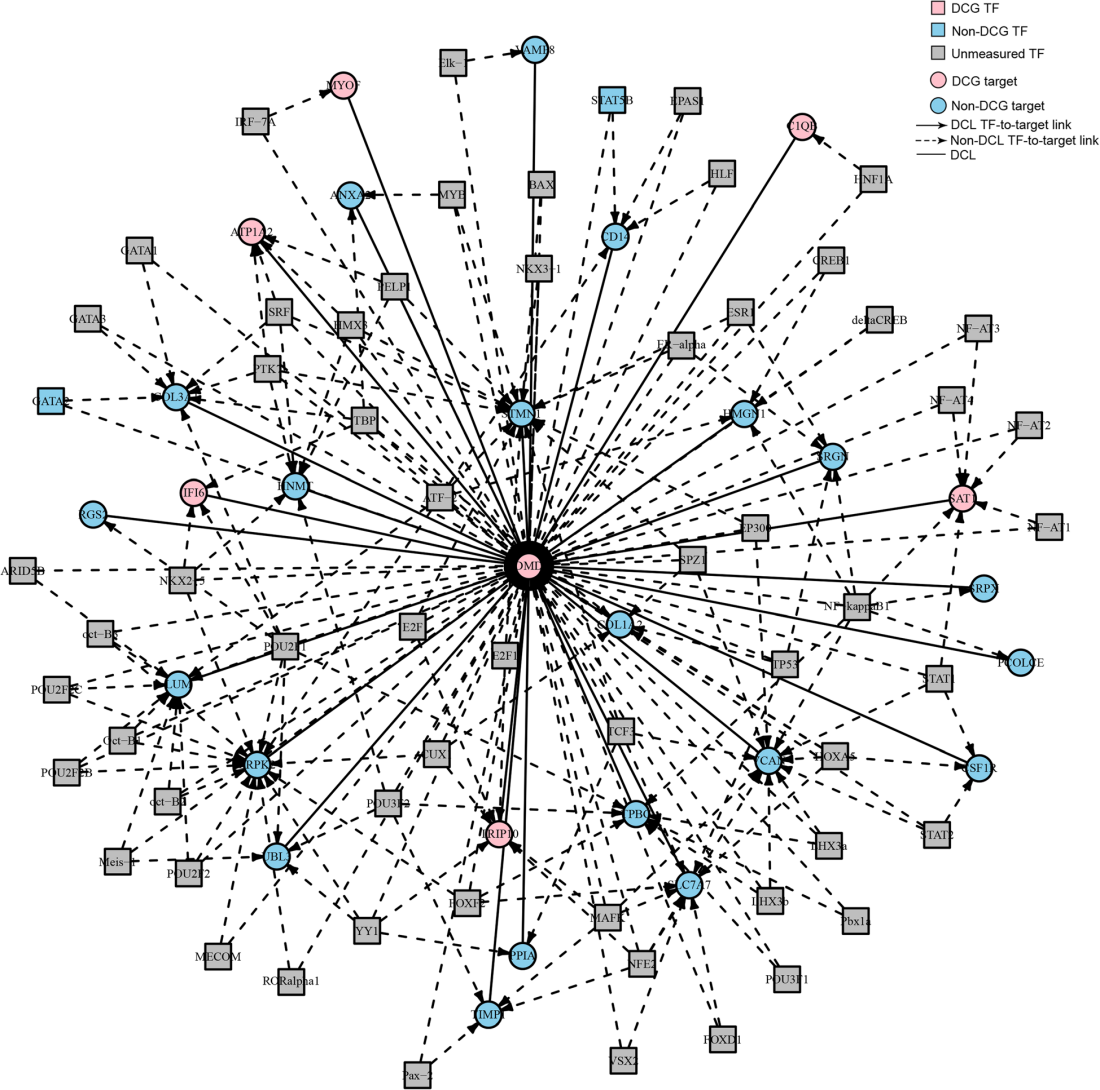
**A subnetwork out of Figure** **2** **surrounding the predefined gene** ***DMD*** **.** TF: Transcription factor; DCG: differential co-expression genes; DCL: links between DCGs.

DCEA results generated 610 pairs of DEGs regulated by at least one common TF. Among these pairs, 78 pairs DEGs were identified as differentially co-expressed. The expression values of a total of 25 pairs of DEGs were positively correlated with each other. A network was generated to illustrate the relationship between the differentially co-expressed genes and their regulatory TFs (Figure 2). Considering the primary causative effect of the aberrant expression of DMD, we construct a subnetwork with *DMD* and its differentially co-expressed genes (Figure 3) to illustrate genes and TFs that may play important roles in the secondary changes of DMD patients. As shown in Figure 3, among the DEGs which shared TFs with *DMD*, six genes were co-expressed with *DMD*, including *ATP1A2*, *C1QB*, *MYOF*, *SAT1*, *TRIP10*, and *IFI6*.

## Discussion

The pathophysiology of DMD is highly complex, involving the dysregulation of many downstream cascades. Gene expression profiling is powerful for investigating the secondary changes of DMD patients. However, previous studies mainly focused on individual gene expression changes, without considering the gene co-expression pattern or the TF information. In this study, with combined two DMD microarray datasets, we implemented DCEA and constructed a TF regulated network with the hope to provide new understanding of the pathogenesis.

Both KEGG pathway and IPA canonical pathway analysis of the DEGs revealed that pathways enriched with aberrantly regulated genes are mostly involved in the immune response processes. This may be due to the infiltration of immune cells into the muscles and the elevated levels of various inflammatory cytokines [18-20]. In addition, the calcium signaling and nNOS signaling in skeletal muscle cells pathway were found to be enriched with deregulated genes, which is consistent with previous findings [3,4].

DCEA results generated 610 pairs of DEGs regulated by at least one common TF, including 78 pairs of co-expressed DEGs. As shown in Figure 2, many TFs are involved in the regulation of these DEGs. Since *DMD* is the causative gene, a subnetwork was constructed to illustrate important genes and TFs that may play important roles in the secondary changes of DMD patients (Figure 3). Among the DEGs which shared TFs with *DMD*, six genes were co-expressed with *DMD*, including *ATP1A2*, *C1QB*, *MYOF*, *SAT1*, *TRIP10*, and *IFI6*. Among them, *ATP1A2*, *MYOF*, and *SAT1* have previously been reported to be involved in muscular dystrophy [21-23]. The relationship between the three left genes and DMD is still known. However, it is worth further investigation. Take *IFI6* for example, this gene was highly expressed in muscle and bone and it is reported to inhibit cytochrome c release from mitochondria by regulating the Ca^2+^ channel which consequently attenuate apoptosis [24]. Alterations in intracellular Ca^2+^ homeostasis are involved in the alteration of apoptosis since a sustained increase in cytosolic Ca^2+^concentration accompanies with apoptosis in cells [25]. Previous study [26] have demonstrated the high influx of extracellular calcium through a dystrophin-deficient membrane, which may lead to subsequent muscle necrosis or apoptosis along with the inflammation response. Whether this gene contributes to the pathogenesis through its regulation of the Ca^2+^ channel needs further investigation. Among the TFs detected in the array, several TFs of *DMD* are dysregulated, including *GATA2* and *STAT5B*. Further studies are needed to investigate their involvement in the disease and the mechanisms of other unmeasured TFs (Figure 3).

The ultimate cure for DMD will lie in the stable introduction of a functional dystrophin gene into the muscles of DMD patients, however, when gene therapy or transplantation of stem cells/ muscle precursor cells will be clinically available is unpredictable. In the interim, reducing secondary features of the pathologic progression of dystrophin deficiency could improve the quality and length of life for DMD patients. Dysregulation of the genes which were co-expressed with DMD may be caused by the malfunction of DMD. Therapeutic strategies aimed to compensate for the dysregulation of these genes may help to reduce the secondary features of DMD. Therefore, genes co-expressed with DMD we identified here may be considered as therapeutic targets in further investigations to treat the secondary effects. In addition, TFs that regulated these genes and DMD may also serve as potential therapeutic markers in future studies.

## Conclusions

In summary, with combined microarray data from the GEO database, we conducted DECA and acquire the TF information of co-expressed DEGs to capture pathogenic characteristics of DMD. Our results may provide a new understanding of DMD and further contribute potential targets for future therapeutic tests.
